# Synthesis and Characterization of New Nanohybrids Based on Carboxymethyl Scleroglucan and Silica Nanoparticles

**DOI:** 10.3390/nano14060499

**Published:** 2024-03-10

**Authors:** Rubén H. Castro, Laura M. Corredor, Isidro Burgos, Sebastián Llanos, Camilo A. Franco, Farid B. Cortés, Eduardo A. Idrobo, Arnold R. Romero Bohórquez

**Affiliations:** 1 Grupo de Investigación en Fenómenos de Superficie—Michael Polanyi, Facultad de Minas, Universidad Nacional de Colombia—Sede Medellín, Medellín 050034, Colombia; caafrancoar@unal.edu.co (C.A.F.); fbcortes@unal.edu.co (F.B.C.); 2 Centro de Innovación y Tecnología—ICP, Ecopetrol S.A., Piedecuesta 681011, Colombia; laura.corredor@ecopetrol.com.co (L.M.C.); aidrobo@yahoo.com (E.A.I.); 3 Grupo de Investigación en Química Estructural (GIQUE), Escuela de Química, Universidad Industrial de Santander, Bucaramanga 680002, Colombia; isidroburgosortiz@gmail.com (I.B.); sllanosg@unal.edu.co (S.L.); arafrom@uis.edu.co (A.R.R.B.)

**Keywords:** scleroglucan, carboxymethyl-scleroglucan, carbodiimide coupling, nanohybrid, EOR

## Abstract

In this study, two new nanohybrids (NH-A and NH-B) were synthesized through carbodiimide-assisted coupling. The reaction was performed between carboxymethyl-scleroglucans (CMS-A and CMS-B) with different degrees of substitution and commercial amino-functionalized silica nanoparticles using 4-(dimethylamino)-pyridine (DMAP) and N,N′-dicyclohexylcarbodiimide (DCC) as catalysts. The morphology and properties of the nanohybrids were investigated by using transmission (TEM) and scanning electron microscopy (SEM), electron-dispersive scanning (EDS), attenuated total reflection-Fourier transform infrared spectroscopy (ATR-FT-IR), X-ray photoelectron spectroscopy (XPS), powder X-ray diffraction (XRD), inductively coupled plasma atomic emission spectroscopy (ICP-OES), thermogravimetric analysis (TGA), differential scanning calorimetry (DSC), and dynamic light scattering (DLS). The nanohybrids exhibited differences in structure due to the incorporation of polyhedral oligomeric silsesquioxane (POSS) materials. The results reveal that hybrid nanomaterials exhibit similar thermal properties but differ in morphology, chemical structure, and crystallinity properties. Finally, a viscosity study was performed on the newly obtained nanohybrid materials; viscosities of nanohybrids increased significantly in comparison to the carboxymethyl-scleroglucans, with a viscosity difference of 7.2% for NH-A and up to 32.6% for NH-B.

## 1. Introduction

Polysaccharides are polymers of monosaccharides bound via o-glycosidic linkages. Their physical, surface, and interfacial properties depend on differences in their conformational parameters, composition, purity, degree of branching and polymerization, and molecular weight [1]. Polysaccharides offer various primary structures and conformations, making them suitable for different applications [2].

Polysaccharides such as xanthan gum, scleroglucan, hydroxyethylcellulose, carboxymethylcellulose, welan gum, guar gum, and schizophyllan [3] have been evaluated as viscosifying additives for enhanced oil recovery (EOR) processes since the mid-1990s [4]. Several experimental and pilot tests have been carried out with biopolymers [5], which have shown increments in oil production compared to water or synthetic polymers such as partially hydrolyzed polyacrylamide (HPAM) [6]. These polysaccharides are good candidates for EOR applications because they are environmentally friendly [7] and most of them are resistant to salinity, temperature, and shear [8]. However, their main disadvantage is their high susceptibility to biodegradation and oxidation [9,10].

Nanopolymer flooding has gained attention in the last decade because nanoparticles (NPs) can improve polymers’ thermal, chemical, and mechanical stability [11,12,13,14,15,16,17]. In addition, NPs have shown the ability to enhance oil recovery by changing the wettability of porous media and reducing the oil–water interfacial tension [18,19,20,21,22,23,24,25]. Two methods are used to incorporate NPs in polymers: (I) the NPs can be dispersed in the polymer solution, and (II) the polymer chains can be covalently bonded to the NPs’ surface. In method I, the NP–polymer interaction occurs through hydrogen bonding, electrostatic binding, ion binding, or hydrophobic forces. In method II, the polymer grafting can be accomplished by covalently attaching a preformed polymer to the NP surface (grafting to) or by in situ polymerization of monomers on the NP surface (grafting from) [26]. The “grafting to” technique is experimentally simple because functional groups of the polymer chains, such as thiol or carboxylic acid, can react directly with NPs or with coupling agents on the NPs’ surface (e.g., silane, titanate, and zirconate) [27]. The most used coupling agents are the silanes with an RSiX3 structure, where X denotes the hydrolyzable groups that react with the -OH groups on the NP surface. R corresponds to the nonhydrolyzable organic group, which reacts with the polymer.

**Figure 1:**
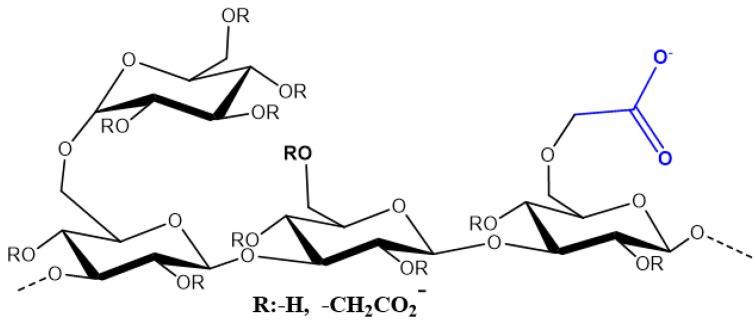
Chemical structure of carboxymethyl-scleroglucan.

Our previous work [28] assessed the effect of the preparation method and the three nanoparticles, namely SiO_2_, Al_2_O_3_, and TiO_2_, on the viscosity and stability of scleroglucan (SG) nanofluids. According to the results, incorporating all NPs improved the viscosifying power of the SG solution due to the formation of NP–SG three-dimensional structures. However, not all nanofluids exhibited colloidal stability. For this reason, in this study, two carboxymethyl-scleroglucan/SiO_2_ nanohybrids were synthesized using the “grafting to” technique. The detailed synthesis of the carboxymethyl-scleroglucan used in this work (Figure 1) has been previously reported by the authors [29]. An *O*-alkylation reaction was performed to insert the monochloroacetic acid hydrophilic group (structure in blue color in Figure 1) in the SG (structure in black color in Figure 1), specifically into the SG’s anhydroglucose units (AGUs). Two carboxymethyl derivatives of SG (CMS) with different degrees of substitution (0.22 for CMS-A and 0.44 for CMS-B) were obtained by changing the amount of sodium bicarbonate used in the reaction. The amide bond formation between the amino-functionalized nanosilica and both carboxymethyl-scleroglucans was mediated by a carbodiimide.

These hybrid materials provide access to highly reactive groups that lead to the preparation of amides of different kinds by direct condensation of existing carboxyl groups [30]. New materials are obtained by transforming the base biopolymer with inorganic materials containing amine-selective groups [31]. Carbodiimide-mediated couplings are commonly used to prepare amides [32,33]. However, in some cases, adding DMAP to the reaction mixture in conjunction with DMAP improves the coupling efficiency. It is more beneficial for those steps involving steric hindrance between the reaction partners. This was the case with a highly branched polymer and a functionalized nanoparticle.

Different characterization techniques were employed to evaluate nanohybrids’ morphological and structural properties (NH-A and NH-B). Finally, the viscosity and thermal stability of both CMS and nanohybrids were compared to determine the effect of the degree of substitution and nano silica content on these properties. To our knowledge, no investigations in the literature report hybrid materials with the same properties as those described in this study.

## 2. Materials and Methods

### 2.1. Materials

The carboxymethyl derivatives of SG, namely CMS-A and CMS-B, were synthesized as described in previous reports [29]. The chemicals employed for synthesizing the nanohybrids NH-A and NH-B were N,N′-Dicyclohexylcarbodiimide (DCC, PM: 206.33 g/mol, ≤100%, Sigma-Aldrich, St. Louis, MO, USA), 4-(Dimethylamino)-pyridine (DMAP, PM: 122.17 g/mol, ≤100%, Sigma-Aldrich, USA), tetrahydrofuran (THF, PM: 72.11 g/mol, ≤100%, Supelco^®^, Bellefonte, PA, USA), and 2-propanol (CH_3_CH(OH)CH_3_, PM: 60.1 g/mol, ≤100%, Supelco^®^, USA). The amino-functionalized nanoparticles used were silicon oxide coated by (3-Aminopropyl)triethoxysilane (SiO_2_ APTES, 99+%, 20 nm, 120 m^2^/g, amphiphilic) manufactured by Nanostructured & Amorphous Materials, Inc. (Los Alamos, NM, USA).

### 2.2. Amidation Reaction

**Figure 2:**
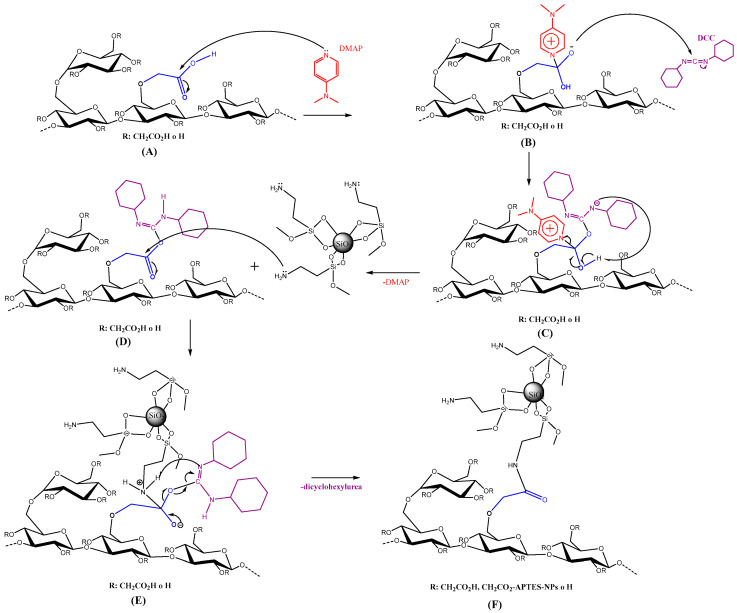
The sequential procedure for synthesizing the nanohybrids (**A**–**F**). Proposed reaction mechanism to obtain NH-A and NH-B.

The amidation reaction was conducted following the method proposed by Valeur-Bradley [34] and Moraillon et al. [35]. First, 1 g of N,N′-dicyclohexylcarbodiimide (DCC) was dissolved in 10 mL of tetrahydrofuran (THF), and the solution was stirred in a beaker for 10 min at 400 rpm. Then, 1 g of CMS-A or CMS-B was added to the solution. The sample was mixed for 10 min at 400 rpm and 30 °C. In another beaker, 0.59 g of 4-(dimethylamino)-pyridine (DMAP) was dissolved in 10 mL of THF by stirring the sample for 10 min at 400 rpm. Then, 0.1 g of SiO_2_ APTES NPs was slowly added to the solution. Finally, both solutions were mixed using an IKA magnetic stirrer and a Teflon-coated magnet (IKA™, Shanghai, China). This reaction lasted 48 h at 400 rpm and 30 °C. The solid was precipitated and washed with THF and isopropanol to remove the unreacted reagents (3 times × 20 mL of each solvent). According to Meiser et al. [36], the nanohybrids were dried under reduced pressure in a rotary evaporator at 0 mBar. In total, 2.22 g and 2.48 g of NH-A (from CMS-A) and NH-B (from CMS-B) powder were obtained, respectively. Figure 2 shows the sequential procedure for synthesizing the nanohybrids where (A) the pyridyl nitrogen of a single DMAP molecule (structure in red color in Figure 2A) attacks the electrode center of the carboxymethyl carbonyl. Subsequently, this electronic delocalization involves (B) the successive reaction of the carboxylate substituents with the DCC (structure in purple color in Figure 2B). Then, by electronic rearrangements, (C) the DMAP catalyst is regenerated, and (D) the O-acyl urea is formed. This intermediate can generate several products; one of them is N-acylurea, which is limited or restricted by the presence of the successive step. This step occurs by directly coupling with the amine of the functionalized nanoparticle (structure in gray color in Figure 2D) to generate (E) a second intermediate [35]. The second intermediate eliminates a by-product formed of dicyclohexylurea (DCU), which was eliminated by washing the sample [37]. Finally, (F) the NH-A or NH-B nanohybrids are obtained [26,33,36,37,38,39].

### 2.3. NH-A and NH-B Characterization

A Bruker Tensor 27 FTIR spectrophotometer (Alpha, Bruker, Billerica, MA, USA) was used to characterize the structure of all samples. Spectra were obtained in the wavenumber range of 4000–600 cm^−1^ using an attenuated total reflection platinum cell (ATR). The data were analyzed using Bruker OPUS 7.5 software.

The simultaneous thermogravimetry–differential scanning calorimetry (STA/TG-DSC) analysis of the nanohybrids was performed by using a Thermal Analysis System TGA/DSC 3+ (STA, Mettler Toledo, Urdorf, Switzerland). The STA curves were analyzed using STAR^e^ software (version 16.00). For each sample, 5 mg was heated from 30 to 1000 °C at a heating rate of 5 °C/min under a nitrogen atmosphere.

Scanning electron microscopy (SEM) and energy-dispersive spectroscopy (EDS) were used to examine the morphology and elemental composition of the nanohybrids. SEM analysis was performed in a Scios2 scanning electron microscope equipped with a Bruker QUANTAX 200 Energy Dispersive X-ray Spectrometer with XFlash^®^ 5010 detector (EDS, Bruker, BER, Germany) with a resolution of 129 eV. EDS spectra were collected using a working distance of 10 mm and an accelerating voltage of 15 kV for 3 min live time. An energy-dispersive spectroscope (EDAX Apolo X, Ametek, Inc., Berwyn, PA, USA) with a resolution of 126.1 eV was used to determine the elemental composition of the nanohybrids.

Transmission electron microscopy (TEM) was performed to determine the size, shape, and structure of both nanohybrids by using a transmission electron microscope (Tecnai F20 Super Twin TMP, Hillsboro, OR, USA) equipped with Gatan US 1000XP-P camera (Pleasanton, CA, USA). TEM samples were prepared by placing a drop of the nanohybrids dispersed in ethanol (200–250 μg/mL) onto a 200-mesh lacey copper TEM grid (400C-FC, Electron Microscopy Sciences, Hatfield, PA, USA) and allowing it to dry at room temperature for 1–2 h. The images were processed in ImageJ software 1.54d (National Institutes of Health, Bethesda, MD, USA) to analyze the particle size distribution. For each sample, over 20 measurements were performed to obtain better statistics.

A D-8 Advance A25 X-ray diffractometer (D8 Advance, Bruker, MA, USA) was used to record X-ray diffraction (XRD) patterns, using a Cu Kα anode operating at 40 kV and 40 mA. The diffraction patterns were acquired at 25 °C across an angular range spanning from 2° to 70°. To conduct the measurements, the samples were pressed between two glass slides on a flat sheet.

Inductively coupled plasma atomic emission spectroscopy (ICP-OES, Optima 8300 ICP-OES Spectrometer, Perkin Elmer, Waltham, MA, USA) was used to determine silicon in the nanomaterials. The measurements were performed at 251.611 nm. The spectral intensity corresponds to the mean of triplicate measurements.

X-ray photoelectron spectroscopy (XPS) was performed in an X-ray photoelectron spectrometer with a PHOIBOS 150 1D-DLD analyzer (NAP-XPS, SPECS Group, Berlin, Germany) using a monochromatic Al-Kα source (1486.7 eV, 13 kV). High-resolution spectra were acquired using a pass energy of 20 eV and a 0.1 eV step. General spectra were obtained using a pass energy of 86.5 eV and 1 eV step. For the high-resolution spectra, 20 measurement cycles were performed, and for the general spectra, 5 cycles were performed. Charge compensation was employed during data collection (3 eV, 20 µA electrons). The samples were mounted on stainless steel metal holders using copper conductive tape. CasaXPS software (version 2.3.25) was used to fit the XPS spectra (Casa Software Ltd., Teignmouth, UK) using the SPECS Prodigy-ACenteno library with the response sensitivity factor established by the manufacturer. A Shirley background was employed. The binding energy scale was calibrated based on the hydrocarbon C 1s peak at 284.8 eV (C-(C, H) component).

The hydrodynamic size of the nanohybrid solutions was measured using a Zetasizer Nano ZS 90 (Malvern Instruments Ltd., Malvern, UK). The reported values of the measurements at 25 °C correspond to the root-mean-square deviation of triplicate measurements.

The viscosities of the nanohybrids and carboxymethyl-scleroglucans solutions were measured in a DV3T viscometer (Brookfield Ametek, Middleborough, MA, USA) at 30 °C and 7.3 s^−1^. The accuracy of the reported value remained at ±1 according to a standard reference.

### 2.4. SG and Nanohybrid Solution Preparation

All solutions were prepared at 1000 ppm as proposed by Abraham and Sumner [37] and Castro et al. [38]. The desired amount of SG, CMS-A, CMS-B, NH-A, or NH-B powder was added to deionized water (DIW) under mechanical stirring (500 rpm). Afterwards, each solution was heated at 40 °C and stirred at 800 rpm for 10 min. Lastly, each solution was mixed at 20,000 rpm for 5 min with a T 25 Digital Ultra-Turrax (IKA™, China).

## 3. Results and Discussion

### 3.1. NH-A and NH-B Characterization

#### 3.1.1. ATR-FTIR Results

**Figure 3:**
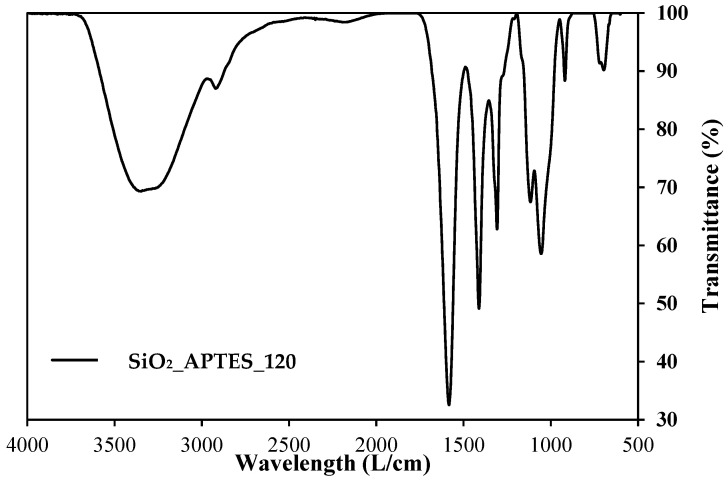
IR spectra of SiO_2__APTES_120.

**Figure 4:**
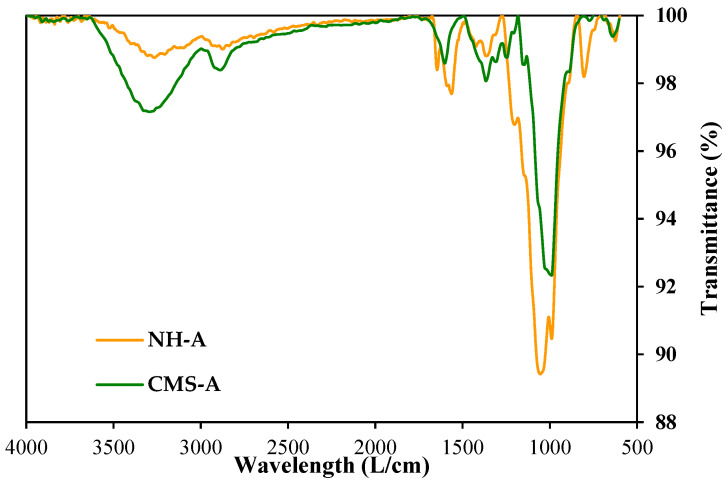
IR spectra of CMS-A and NH-A.

**Figure 5:**
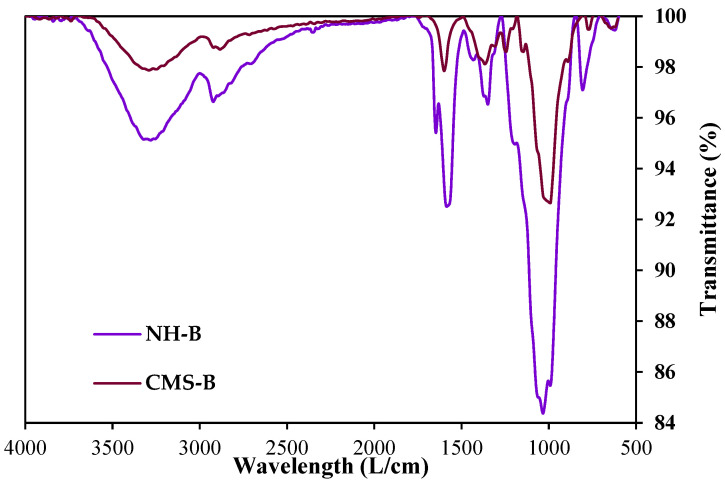
IR spectra of CMS-B and NH-B.

Figure 3 presents the IR spectra of the amino-functionalized silica nanoparticles (SiO_2__APTES_120). Figure 4 shows the IR spectra of the CMS-A and NH-A, and Figure 5 displays the IR spectra of the CMS-B and NH-B. A peak with low intensity is detected at 1600 cm^−1^ in the CMS-A and CMS-B spectra, corresponding to the bending vibration of the OH groups [29,40,41] due to the vibrational stretching of the carboxyl functional group or carboxy group (-COOH). This peak was transformed for the NH-A and NH-B in two new signals as a doublet with intensities at 1645 cm^−1^ and 1560 cm^−1^, corresponding to one of the signals of an amide II, the first one being attributed to the C=O stretching vibration in combination with a -NH deformation vibration, respectively. Around 766 cm^−1^ [42], the peaks of the in-plane vibrations of branched aliphatic carboxylic acids in the fingerprint region disappeared, and the Si-C stretching signals attributed to the -O-Si-CH_2_-C siloxanes of linear polymers or to SiO_2_ silica signals appeared [43].

#### 3.1.2. STA/TG-DSC Results

**Figure 6:**
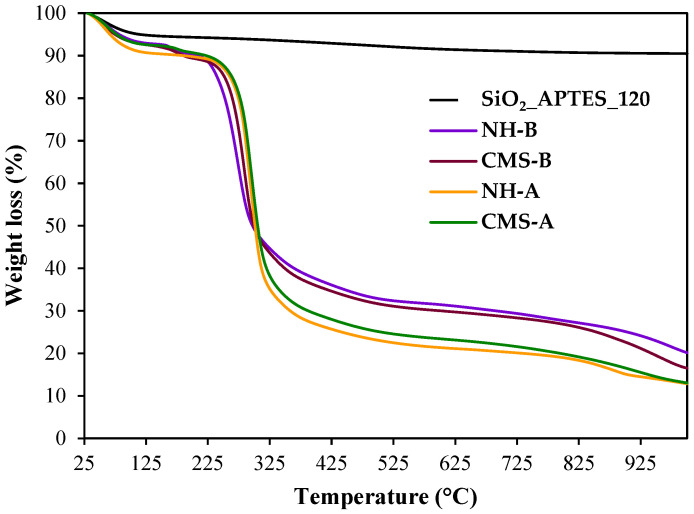
TG curves of SG, CMS-A, CMS-B, NH-A, and NH-B under nitrogen atmosphere.

Figure 6 and Table 1 present the TG and DTG behavior of the SG, CMS-A, and CMS-B [29] and compare them with the results for both nanohybrids (NH-A and NH-B) and the nanoparticle used for their synthesis.

In its first transformations, the NH-A exhibits scleroglucan-like behavior, with four stages of weight loss. The first stage is observed below 100 °C and is assigned to the loss of water. The second weight loss (above 122 °C with a DTG peak at 57 °C) can be associated with the decomposition of the pyranose ring structure. The weight loss in this stage was 59% [44,45].

On the other hand, five stages of weight loss are observed in the NH-B. The first stage is between 30 and 141 °C, ascribed to the loss of water. The second one occurred above 142–193 °C with a 2% weight loss, related to the decomposition of the traces of unreacted carboxylic acid groups [46]. The third stage is observed between 201 °C and 388 °C, with a 53% weight loss, and it is related to the decomposition of the carboxylate and amide groups [47]. The four stages occurred between 389 °C and 800 °C with a 10% weight loss, corresponding to the breakdown of the C-C bonds within the biopolymer. The last stage, with 8% weight loss, was observed from 801 to 999 °C and was attributed to the product residue. The NH-B is more stable than the NH-A due to its higher silica nanoparticle content.

Finally, the nanoparticles’ thermogram revealed two weight loss stages with 5% weight loss each. The first occurred up to 139 °C, corresponding to the adsorbed water. The second drop (139–900 °C) is attributed to the decomposition of the aminopropyl groups. Finally, 90% of the residue of the inorganic material (silica) remained.

#### 3.1.3. SEM-EDS Analysis

**Figure 7:**
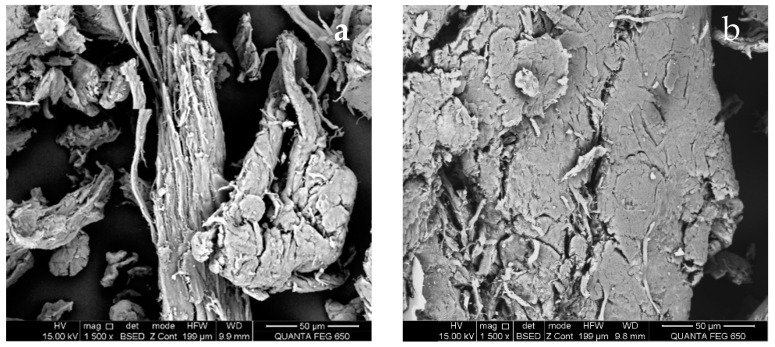
SEM micrographs of (**a**) CMS-A at 1500× and (**b**) CMS-B at 1500×.

Micrographs were obtained using the backscattered electron detector (BSE). Figure 7 shows the SEM micrographs of CMS-A and CMS-B. According to Castro et al. [29], the CMS-A (Figure 7a) has a less fragmented fibrillar structure than CMS-B (Figure 7b).

Contrasts can be seen that depend mainly on the average atomic number. Lighter areas in BSE correspond to high average atomic numbers, while the dark ones belong to low atomic numbers.

The type of EDX detector employed allows the detection of all elements of Z ≥ 5 found in the analyzed area. The height of the peaks in the spectra can be interpreted as reflecting the relative abundance of these elements in the evaluated part. A low-magnification image is presented for the samples to show the general morphology; likewise, a magnification is performed in areas of interest with their elemental composition.

**Figure 8:**
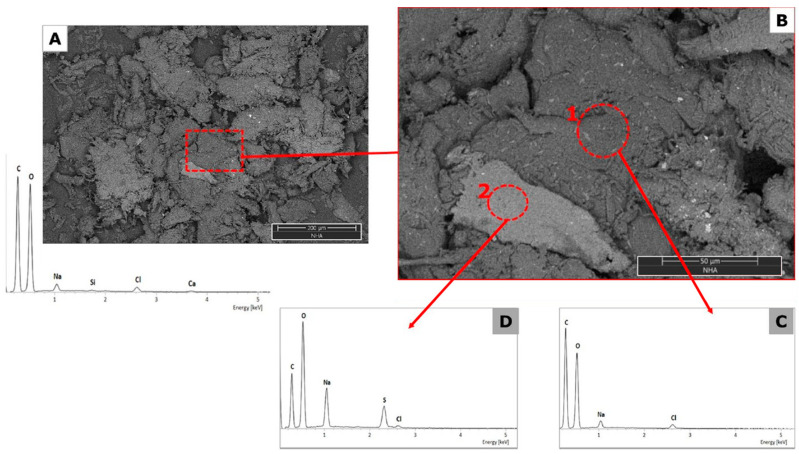
SEM micrographs of NH-A at (**A**) 500×, (**B**) 2000×, (**C**,**D**) EDS spectra.

**Figure 9:**
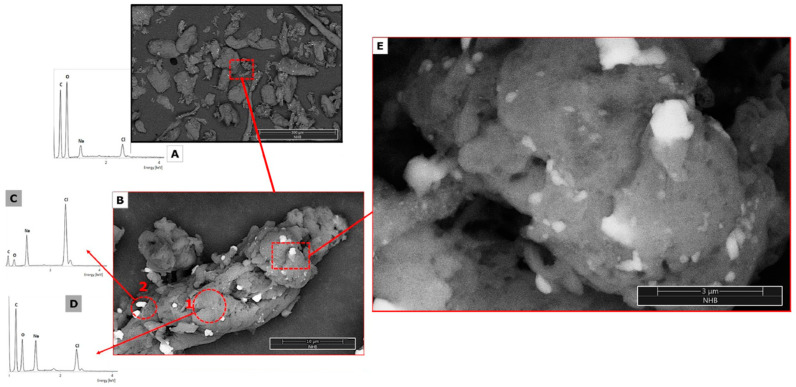
SEM micrograph of NH-B (**A**) 500×, (**B**) 10,000×, (**E**) 35,000×, (**A**,**C**,**D**) EDS spectra.

Figure 8 and Figure 9 show the SEM images of NH-A and NH-B; both nanohybrids have compact fibril structures, suggesting that the amidation reaction altered the CMS materials [33]. The changes in the surface microstructure of CMS suggest their coupling with the nanoparticles to form the nanohybrids NH-A and NH-B. The nanohybrids’ EDS spectra show carbon, oxygen, sodium, chlorine, and calcium content similar to carboxymethyl-scleroglucans [29].

#### 3.1.4. TEM Analysis

**Figure 10:**
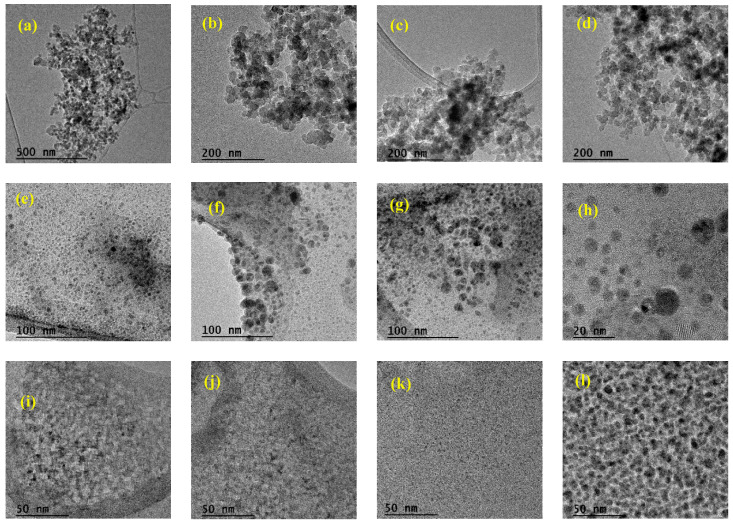
TEM images of (**a**–**d**) SiO_2_-APTES NPs at 19.50K×, 43.00K×, 38.00K× and 38.00K×; (**e**–**h**) NH-A at 97.00K×, 97.00K×, 97.00K×, 285.00K×; and (**i**–**l**) NH-B at 145.00K×, respectively.

Figure 10 shows representative electron microscopy images of the NH-A and NH-B nanohybrid obtained by a one-step synthesis method. The commercial nanoparticles are agglomerated spheres with an average size of 22 nm.

The NH-A and NH-B products’ micrographs show a bright contrast covering a large area, attributed to the biopolymer, and a dark contrast assigned to the spherical nanoparticles.

The NH-A micrographs show spheres with an average size of 12.2 nm with diameters between 8 nm and 18 nm. The area-average random interplanar distance between distant spheres is 19 ± 6 nm, and 1.5 ± 0.6 nm between the closest ones. Likewise, the NH-B presents an average size of 8.0 nm. It has an area-averaged random interplanar distance of 4.5 to 1.6 nm between all adjacent spheres.

The micrographs show the differences between both nanohybrids, showing a higher number of nanoparticles per area on the NH-B than the NH-A. This indicates that the degree of substitution of the CMS-A and CMS-B changes the active sites available for the covalent bonding with the amino-functionalized nanoparticles and, for instance, the properties of the nanohybrid synthesized.

#### 3.1.5. X-ray Diffraction (XRD) Analysis

**Figure 11:**
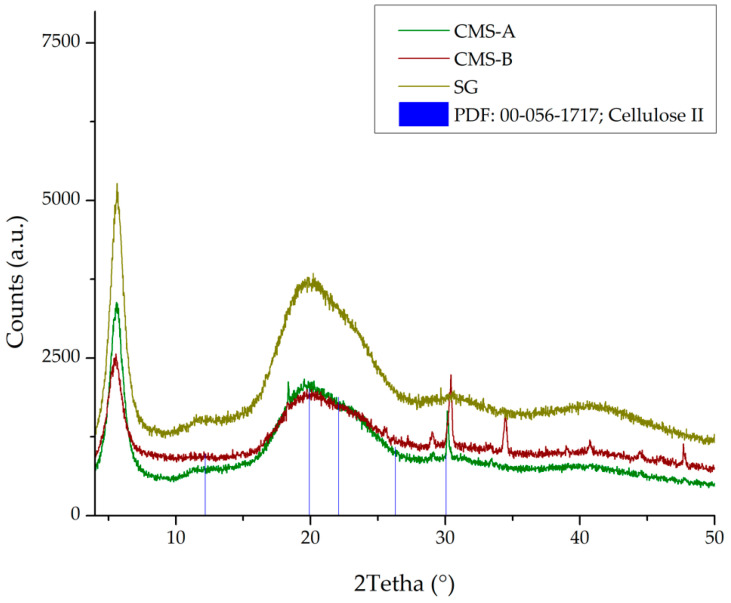
XRD patterns of CMS-A, CMS-B, SG, and Cellulose II.

The CMS-A, CMS-B, and SG patterns (Figure 11) were compared with crystalline cellulose II (diffraction pattern file PDF 00-056-1717) [48]. As reported in our previous work [29], SG, CMS-A, and CMS-B show a broad halo peak centered at 2theta value of 20° due to their amorphous structure [49]. A partial arrangement of the SG structure, attributable to the amidation reaction, is observed in the peaks at 2tetha value around 30°.

**Figure 12:**
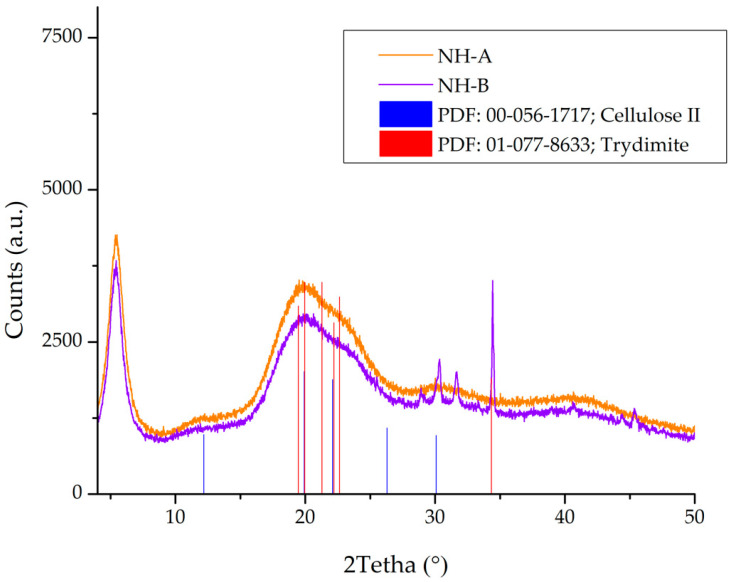
XRD patterns of NH-A, NH-B, Cellulose II, and Trydimite.

Figure 12 shows the powder patterns of the NH-A and NH-B materials and the correlation of these materials with the reported powder pattern of the diffractogram of crystalline cellulose II and tridymite (identified with the diffraction pattern file PDF 01-077-8633). Differences between the 2theta values of 30–40° can be seen due to different functional groups in these materials. The most intense reflection is observed near the 2theta value of 34°. The cellulose-type nature of the scleroglucan is observed in the NH-A and NH-B. Likewise, the silicon dioxide from the nanoparticle is identified. SiO_2_ is in the form of tridymite that crystallizes in an orthorhombic crystalline system, which coincides with that reported by the supplier of the nanoparticles. A considerable part of these materials is amorphous due to the amorphous nature of the biopolymer and the nanoparticle used for their synthesis.

The differences between the CMS-A and CMS-B patterns and the NH-A and NH-B patterns are attributed to the new functional groups incorporated into the carboxymethyl-scleroglucans after the amidation reaction.

#### 3.1.6. ICP-OES Analysis

Table 2 summarizes the results for CMS-A and CMS-B and their nanohybrids. As expected, the content of SiO_2_ in NH-B is higher than in NH-A due to the higher degree of substitution and higher number of active sites available for the covalent bonding with the amino-functionalized nanoparticles in the CMS-B. This analysis confirms the formation of both nanohybrids.

#### 3.1.7. XPS Analysis

**Figure 13:**
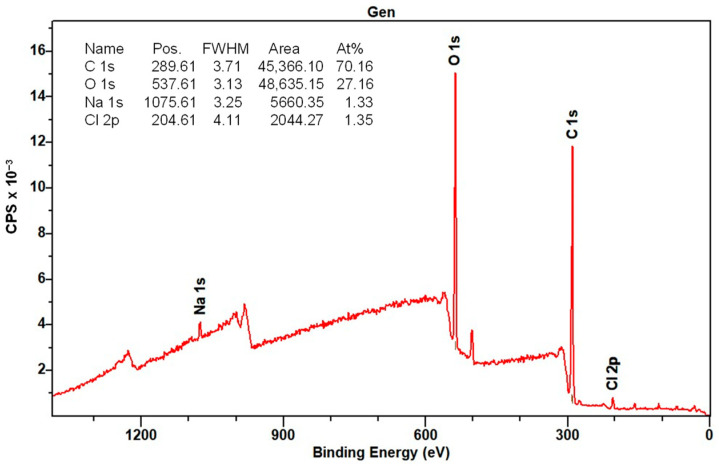
XPS spectra of CMS-B.

The elemental composition of the surface of the CMS-B, NH-A, and NH-B was determined by XPS. Figure 13 shows the CMS-B spectra where the presence of C 1s (289.6 eV), O 1s (537.6 eV), Na 1s (1075.61 eV), and Cl 1s (204.6 eV) can be identified. These elements come from synthesizing the carboxymethyl-scleroglucan with sodium bicarbonate (NaHCO_3_) and monochloroacetic acid (AMCA). The peak intensity indicates a high C (70.1%) and O (27.16%), with an O/C ratio of 0.38.

**Figure 14:**
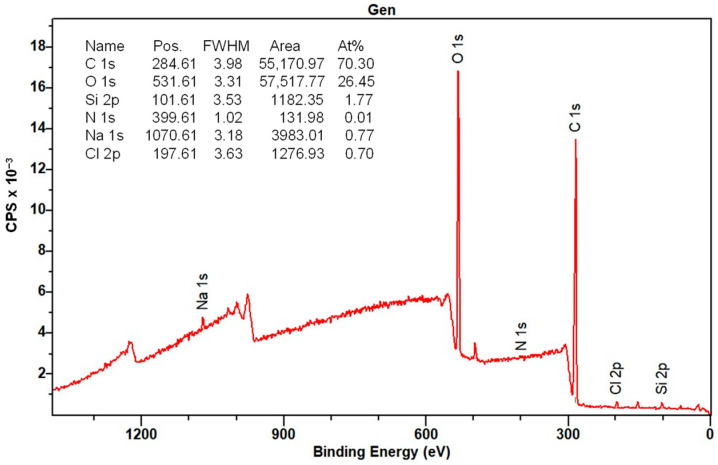
XPS spectra of NH-A.

**Figure 15:**
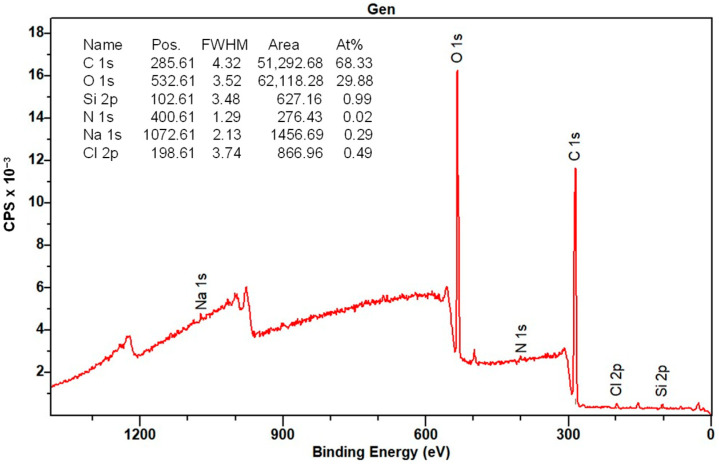
XPS spectra of NH-B.

Regarding the NH-A (Figure 14) and NH-B (Figure 15) spectra, the presence of C 1s (286 eV), O 1s (532 eV), Na 1s (1072 eV), and Cl 1s (198 eV) persists; however, the Si 2p (102 eV) and the N 1s (400 eV) signals appeared and are attributed to the nanoparticles SiO_2__APTES_120 and the amide bond formed in the nanohybrids. This is corroborated by the NIST (National Institute of Standards and Technology, Gaithersburg, MD, USA) database [50,51,52] and with the results from previous studies [32,33,51,52].

The deconvolution of the primary spectra in the regions of high resolution and the central level spectra of C 1s, O 1s, Na 1s, Cl 1s, Si 2p, and N 1s for CMS-B, NH-A, and NH-B are presented in Table 3. The XPS spectra indicate the presence of different functional groups, such as the methyl C ring at 283 eV, the carbon bonds of the cellulosic structure of the SG (C_6_H_10_O_5_) at 284 eV, the carbons and oxygens of the C-O-C glycosidic bonds at 286 eV and 530 eV, respectively, and the carbonyl group incorporated into the structure (C-O and CO=O) at 288–289 eV [51,52]. In addition, the high-resolution XPS spectra of O 1s exhibit the same oxygenated functional groups formed by the carboxymethylation of scleroglucan. These groups are the ether -O*CH_2_C(O)- at 534 eV and the carbonyl -OCH_2_C(O*)- at 532 eV functional groups. Likewise, interference persists from two additional components at 1072 eV and above 200 eV, corresponding to sodium (Na 1s) in NaCl and chlorine (Cl 1s), respectively.

Finally, the presence of Si–O–C in the NH-A and NH-B products at 103 eV [53], which was not identified in the CMS-B spectra, was observed. The XPS results confirm the CMS and SiO_2__APTES_120 hybridization by the presence of C 1s, O 1s, Na 1s, Cl 1s, Si 2p, and N 1s in the regions.

### 3.2. NH-A and NH-B Solution Analysis

#### 3.2.1. DLS Analysis

**Figure 16:**
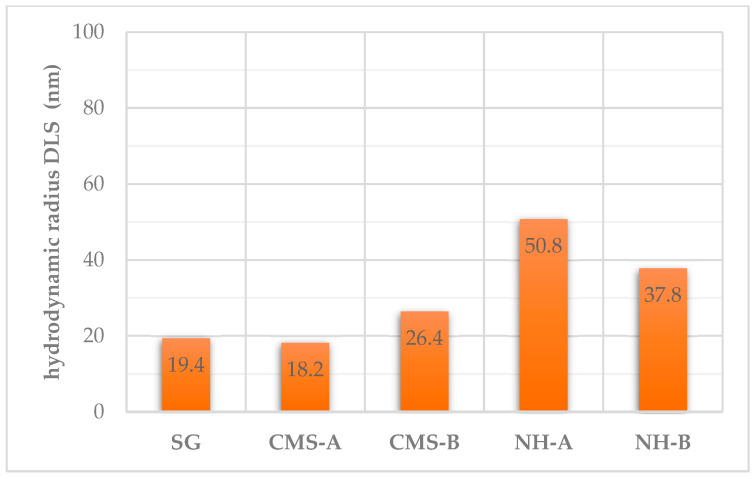
Average hydrodynamic diameter (d-50) of SG, CMS-A, CMS-B, NH-A, and NH-B.

As presented in Figure 16, the hydrodynamic diameter (d-50) of the SG, CMS-A, and CMS-B precursors in water were 19.4, 18.2, and 26.4 nm with a polydispersity index of 0.84, 0.73, and 0.55, respectively. This presents a significant increase in the hydrodynamic diameter due to the carboxymethylation substitution where the structure branches (stretching) are in higher proportion in CMS B concerning scleroglucan. On the other hand, the nanohybrid results are 50.8 nm for NH-A and 37.8 nm for NH-B, with a polydispersity of 0.96 and 0.97, respectively, suggesting the presence of smaller aggregates than CMS products in suspension (shrinking) and more stability with NH-A and NH-B.

#### 3.2.2. Viscosity Measurements

**Figure 17:**
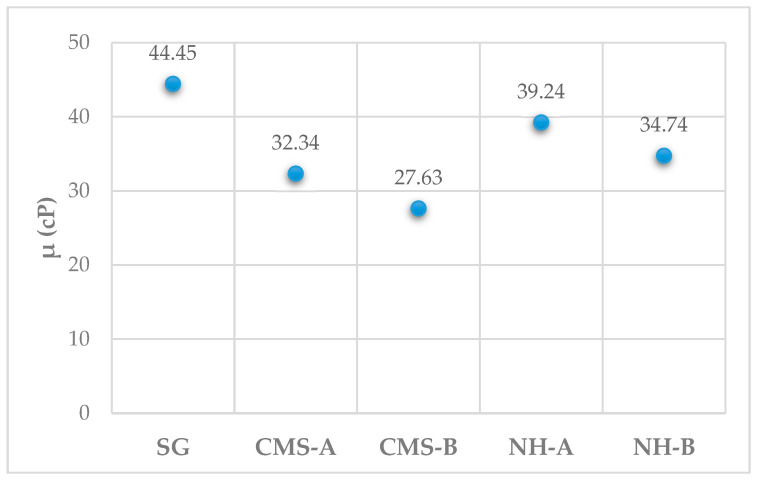
Viscosity of SG, CMS-A, CMS-B, NH-A, and NH-B solutions at 7.3 s^−1^ and 30 °C.

The viscosity of 1000 ppm SG, CMS-A, CMS-B, NH-A, and NH-B solutions are reported in Figure 17. Viscosity increments of 7.2% and 32.6% were observed for the NH-A and NH-B compared to their precursors CMS-A and CMS-B, respectively, which is attributed to the formation of an NP–biopolymer network. On the other hand, the NH-B exhibits lower viscosity values than the NH-A due to a greater breakage of the SG triple helix caused by the higher degree of substitution in the CMS-B. The incorporation of the nanoparticle in the structure of the CMS-A and CMS-B cannot compensate for the breakage of the SG helix. Still, it will probably improve the resistance of the biopolymer to microbial degradation for EOR applications. Further studies will be performed to evaluate this effect.

## 4. Conclusions

Two new nanohybrids (NH-A and NH-B) were synthesized from amide-type covalent bonds between two carboxymethyl-scleroglucans (CMS-A and CMS-B) and commercial amino-functionalized silica nanoparticles using N,N′-dicyclohexylcarbodiimide and 4-dimethylaminopyridine as catalysts.

TEM, XPS, and FTIR analyses confirmed the amidation reaction. According to the SEM-EDS analysis, NH-A and NH-B nanohybrids are heterogeneous solids with compact fibrillar structures, suggesting that the amidation reaction altered the CMS materials. According to the thermal analysis, the nanohybrids’ dehydroxylation temperature was reduced, but the CMS’s thermal stability was maintained below 130 °C and above 300 °C.

In conclusion, the formation of an NP–biopolymer network increased the viscosity values of both carboxymethyl-scleroglucans. Still, it cannot compensate for the viscosity loss of the SG caused by the breakage of its triple helix due to the chemical modifications performed. Additional investigations will be conducted to assess the microbial, mechanical, chemical, and thermal degradation of both types of NH to evaluate their potential as EOR additives.

## Figures and Tables

**Table 1:** TG and DTG parameters.

Sample	T, °C	Weight Loss, %	Endothermic Peaks of DSC, °C	DTG Peaks, °C	MWLR, (% min^−1^)
CMS-A	30–122	7.0	62.9	58	0.80
	123–230	3.0	172.0	177	1.20
	231–521	65.0	240.0	298	4.31
	522–999	12.0	399.8		
	Residue, 1000 °C	13.0			
NH-A	30–122	9.0	57.0	58.2	-
	122–400	69.0	294.0	295	1.40
	401–800	7.0	-	-	-
	801–999	6.0	821	879	-
	Residue, 1000 °C	13.0			
CMS-B	30–122	8.0	54.5	59	0.86
	122–208	2.0	171.8	175	2.42
	209–524	60.0	234.7	285	3.86
	525–999	13.0	396.0		
	Residue, 1000 °C	17.0			
NH-B	30–123	7.0	-	56	-
	124–200	2.0	165	167	2.42
	201–388	53.0	270 (exothermic)	276	0.9
	389–800	10.0	-	-	-
	801–999	8.0	-	-	-
	Residue, 1000 °C	20.0			
SiO_2__APTES_120	30–139	5.0	61.0	57	0.1
	139–999	5.0	767.0; 821 (exothermic)	-	-
	Residue, 1000 °C	90.0			

**Table 2:** Si content by ICP-OES and conversion to SiO_2_.

Sample	Si (ppm)	SiO_2_ (ppm)
CMS-A	0.07	0.15
NH-A	2.66	5.70
CMS-B	0.06	0.12
NH-B	2.84	6.07

**Table 3:** Values of binding energy of the main functional groups.

Energy Level	Functional Groups	CMS-B	NH-A	NH-B	NIST Database
C 1s	C-(CH_2_)	283.19		283.02	283.30
	C-(C)	284.60	284.60	284.60	284.60
	(C)-O	285.33			285.00
	(C)-O-(C)		285.87	286.01	286.00
	O-(CH_2_)	287.00	287.07		287.00
	(C)O=O	288.92	288.05	287.69	288.50
	(C)=O		289.62	289.60	288–290
O 1s	C-(O)-C	530.67		530.76	530.75
	C=(O)	532.03	531.95	532.38	531.5–532
	C-(O)	533.19	533.16	533.83	533.00
	(O)-CH_2_		534.15		534.1
Si 2p	(Si)–O–C		103.37	102.82	102.8
Na 2s	(Na)Cl	1071.66			1071.6
	(Na)Cl		1072.21		1072.2
Cl 2p	Na(Cl)	198.45	198.91	198.06	200
	Cl organic	200.93	200.61	199.57	198.3

## Data Availability

The data presented in this manuscript are available on communication to the corresponding author.
